# Application of the Biorefinery Concept in the Processing of Crambe (*Crambe abyssinica* Hochst) Seed Defatted Meal in a Pressurized Medium

**DOI:** 10.3390/plants14030326

**Published:** 2025-01-22

**Authors:** Camila da Silva, Jefferson Alessandro Schmitz, Djéssica Tatiane Raspe, Natália Stevanato, Jaqueline Hoscheid, Marcelino Luiz Gimenes, Beatriz Cervejeira Bolanho Barros, Lúcio Cardozo-Filho

**Affiliations:** 1 Programa de Pós-Graduação em Engenharia Química, Universidade Estadual de Maringá, Av. Colombo 5790, Maringá 87020-900, PR, Brazil; djessicaraspe@hotmail.com (D.T.R.); natalias.stevanato@gmail.com (N.S.); mlgimenes@uem.br (M.L.G.); lcfilho@uem.br (L.C.-F.); 2 Departamento de Tecnologia, Universidade Estadual de Maringá (UEM), Av. Angelo Moreira da Fonseca 1800, Umuarama 87506-370, PR, Brazil; jefferson.schmitz77@gmail.com (J.A.S.J.); bcbolanhobarros@uem.br (B.C.B.B.); 3 Programa de Mestrado Profissional em Plantas Medicinais e Fitoterápicos na Atenção Básica, Universidade Paranaense, Umuarama 87502-210, PR, Brazil; jaquelinehoscheid@prof.unipar.br

**Keywords:** *Crambe abyssinica* Hochst, dry extract, processed flour, phytochemicals, hydroalcoholic solvent

## Abstract

The valorization of byproducts such as defatted meal (DM) is essential for the implementation of a biorefinery structure and can be achieved through the application of emerging technologies, such as pressurized liquid extraction. This work aimed to apply pressurized liquid extraction to obtain products derived from the DM of crambe (*Crambe abyssinica* Hochst) seeds. The experiments investigated the effect of ethanol percentage in the hydroalcoholic solvent (25%, 50% and 75%, *v*/*v*) on the mass extract yield (MEY) and on the composition of the products obtained: phytochemical extract (PE) and processed flour (PF). The PE obtained using 25% ethanolic solvent had the highest MEY (23.48 wt%) and phenolic compounds, composed of caffeic, gallic and ferulic acids, which conferred activity against the fungus *C. albicans*. The solvents tested did not influence the content of soluble proteins. The solvent with 75% ethanol promoted the highest levels of glucosinolates (258.94 μmol/g) and tannins (8.80 mg/g) in the PE, reducing 96% and 98% of these contents in the PF produced. The PF obtained in the extraction with 75% ethanol contained phenolic compounds (~23 mg/100 g), dietary fibers (54.25 g/100 g) and soluble proteins (26.39 wt%), mainly composed of glutelin fraction. The PF also presented adequate functional properties, such as water solubility and absorption, which suggest potential use in pet food formulations.

## 1. Introduction

Crambe (*Crambe abyssinica* Hochst) is a non-edible oilseed native to the Mediterranean region, which has favorable agronomic characteristics such as a short life cycle, resistance to pests and diseases and relative tolerance to drought [1]. The oil, extracted from the seeds, is mainly used in the production of biodiesel, whose extraction process generates a defatted flour (DM) with phytochemical potential as a byproduct [1,2]. Biorefinery is characterized as the sustainable processing of biomass into a variety of marketable products (such as food, feed, materials and chemicals) and energy. Through biorefinery, it is possible to reduce waste generation, since it is possible to achieve complete use of biomass to obtain high added value products [3]. Waste management and valorization in a biorefinery approach is an important aspect, which encourages the study and development of technologies for the valorization of DM. Therefore, it is essential to investigate sustainable processes that can segregate the active compounds and macromolecules of interest, aiming at maximum use of the by-product based on its applicability in various sectors of highly valued products.

The presence of proteins in the DM composition of crambe seeds has sparked interest in its application in cattle feed [4,5]. Furthermore, phytonutrients such as phenolics and glucosinolates are also reported in the composition of DM [6], therefore extracting these compounds will provide a high-quality phytochemical extract (PE), in addition to generating processed flour (PF). The PE can be used as an ingredient to enrich nutraceutical products, by directly or indirectly incorporating the extracted compounds. The PF can be added to food products, such as bakery products, to increase the protein and/or dietary fiber content, as well as to compose food supplements. However, for application in animal feed, the glucosinolate content in the PF produced must be reduced, since high levels of glucosinolate consumption can cause deleterious effects to animals [7]. Thus, the use of these products, which come from a sustainable process, contributes to the valorization of the raw material, through its full use.

As a strategy for obtaining these products, pressurized liquid extraction (PLE) can be applied, which consists of the simultaneous action of temperature and pressure under conditions sufficient to maintain the solvent in its liquid state. In this methodology, the extraction of compounds is promoted due to the changes caused in the properties of the extracting solvent when it is subjected to the operational conditions of the technique, which allow easy and deep penetration into the matrix, from which the compounds will be extracted, increasing the diffusion rate and solubility of the analytes [8,9]. Due to the operational intensification caused by the process, this technique is an alternative to conventional extraction methods, considered as efficient and fast, with reduced consumption of solvents [10]. In order to promote the application of PLE, the use of non-toxic and environmentally safe solvents should be considered, with ethanol and water mixtures as an option classified as food grade [11]. In addition to having the capacity to extract a wide range of polar compounds [12], hydroethanolic mixtures are cost-effective compared to the use of pure organic solvents. The selectivity of this mixture is easily adapted by increasing or reducing the ethanol concentration, which allows the exploration of a complex matrix and the valorization of active compounds present in its composition [11].

The objective of this work was to investigate the application of PLE to obtain products derived from the DM of crambe seeds. For this purpose, the effect of the composition of the hydroethanolic solvent was evaluated on the mass yield and chemical composition of the PE, as well as its influence on the characteristics and composition of the PF. This study is part of a broad project by the research group that aims to make greater use of unconventional oilseeds by applying the concept of biorefineries and processes in pressurized conditions. The strategy applied in this work is a step after oil extraction from crambe seeds, which will result in the higher added value of the seeds. Three products will be generated from this proposal, considering that vegetable oil was obtained in the first stage developed by Iwassa et al. [13].

## 2. Results and Discussion

### 2.1. Phytochemical Extract (PE)

The data obtained regarding the effect of the solvent composition on obtaining the phytochemical extract (PE) and the characteristics of the PE are presented in Table 1. The run E25 provided greater mass extract yield (MEY), which is explained by the fact that the presence of water in the solvent composition reduces the selectivity of the process and solubilizes a greater range of compounds [14]. Water acts to swell the plant material, increasing the contact surface between solvent and solute in the extraction medium [15]. This effect is desirable when the objective is to obtain higher extraction yields; however, it can lead to significant extraction of highly polar compounds that are not of interest, reducing the quality of the extracts obtained [16].

The composition of the solvent had no effect on the soluble proteins content of the extract, which is associated with the fact that the hydrogen bonds of water are broken under supercritical or subcritical conditions, and therefore water becomes less polar and has properties similar to ethanol [17]. Therefore, increasing or reducing the concentration of ethanol in the solvent does not cause major changes in the solvent properties to the point of influencing protein extraction.

Regarding total glucosinolates, the results obtained showed that the recovery of these compounds was moderately more efficient when a solvent with a higher proportion of ethanol (E75) was used, which is due to the fact that glucosinolates are more soluble in ethanol than in water [18]. Studies report that an ethanol concentration in the range of 60% to 80% is ideal for glucosinolate extraction [19,20,21]. The application of higher concentrations of ethanol may lead to protein coagulation and polysaccharide precipitation, reducing the extractability of glucosinolates [22]. Pagliari et al. [21] investigated predictive bioactivities of purified glucosinolates extract and reported the high probability of chemotherapeutic activity as well as the chemopreventive and antineoplastic effects of these compounds.

The tannin content of the extract increased as the polarity of the solvent decreased. In this study, the tannins determined in the extracts are in condensed form, since the method employed involves a condensation of vanillin with catechin in an acidic medium. Zanchi et al. [23] reported that the solubility of tannins in water is a decreasing function of the degree of polymerization and therefore condensed tannins (obtained by the polymerization reaction) are more soluble in solvents with a higher proportion of ethanol.

It is noted that the sucrose content in the extract increased when the ethanol concentration in the solvent was increased from 25% to 50%. In the extraction process using hydroalcoholic solvent under subcritical conditions, sucrose hydrolysis may occur and the hydrolysis rate is reduced with increasing ethanol concentration [24], which may justify the higher sucrose content in the extract from E50. However, the increase in ethanol concentration (E75) reduced the sucrose content in the extract, which may be associated with the drastic reduction in solvent polarity, decreasing the solubility and extractability of the sugar.

Regarding the phenolic compounds present in the PE composition, six compounds were identified and quantified, comprising five phenolic acids and one flavonoid. It is known that the solubility of the compounds in an extraction process depends mainly on the interactions with other components and the polarity of the solvent [25]. Especially in the latter case, the increase in solvent polarity, associated with the reduction in solubility of the mentioned compounds, which are predominantly hydrophilic, resulted in a reduction in their removal from defatted meal (DM). For example, gallic acid is extracted more effectively using solvents with a polar predominance [26]. This can be verified in terms of phenolic acids, where 421.43, 389.73 and 275.50 mg/100 g accounted for the total constituents in the PEs resulting for E25, E50 and E75, respectively. Caffeic acid was the main component in the analyzed extracts, and despite the predominant sequence to the composition of the PE resulting from E25 (caffeic acid > gallic acid > ferulic acid) as different from the PEs resulting from E50 and E75 (caffeic acid > ferulic acid > gallic acid), all of these substances correspond to the class of phenolic compounds responsible for providing antioxidant activity to the extract, which can provide it with a biological effect [27].

Quercetin extraction, on the other hand, was favored by solvents with a higher amount of ethanol. It is known that this biological compound is characterized as a polar substance; however, DM is a complex matrix and presents different compounds with varied properties, thus the results do not follow a solubility trend. In addition to this, this flavonoid constitutes the class of polyphenols, which in plant matrices are generally linked to polysaccharides or proteins [28]. Therefore, it is possible that during extraction there was interference from some of these compounds, since the other PE components also do not describe integral behavior (Table 1).

The antimicrobial activity of the extracts was evaluated in terms of MIC and MBC and the results were presented in Table 2. It is noted that the bacteria evaluated (*E. coli* and *S. aureus*) showed less resistance to the extracts from E25 and E75, while the fungus (*C. albicans*) was more sensitive to the extract obtained from E25 and E50. Furthermore, it was found that the extract obtained at E50 did not present a bactericidal effect and the other extracts presented MBC > 250 mg/mL, regardless of the bacteria investigated. All extracts present MBC = 125 mg/mL against *C. albicans*.

The antibacterial and anticandidal effects found in the extracts can be attributed to the composition of glucosinolates, tannins and phenolic acids. Njokuocha [29] reported that tannin extract from Vitex doniana leaves had an MIC of 1.25, 2.5 and 0.3125 mg/mL against *E. coli*, *S. aureus* and *C. albicans*, respectively. Isothiocyanates, products of glucosinolate hydrolysis, show MIC of 0.10 mg/mL against *E. coli* and *S. aureus* [30]. Caffeic acid inhibited *C. albicans* in forming biofilm (MIC = 0.25 mg/mL) [31], in addition to inhibiting the growth of *E. coli* (MIC = 7.05 mg/mL) and *S. aureus* (MIC = 3.50 mg/mL) [32]. Ferrulic acid showed MIC of 0.25 mg/mL against *E. coli* and *S. aureus* and 0.13 mg/mL against *C. albicans* [33]. Strains of the same fungus were tested against isolated gallic acid and were found to be susceptible to this phenolic acid (MIC = 0.32 mg/mL) due to changes in membrane integrity and mitochondrial potential [34]. Quercetin also exerted inhibitory effects against *S. aureus* (MIC = 0.02 mg/mL) and *E. coli* (MIC of 4.00 mg/mL) [35].

Thus, the greater antifungal activity of the extracts obtained in E25 and E50 is possibly associated with the higher content of phenolic acids found in these extracts. On the other hand, the results suggested that the antibacterial activity may have been influenced by the other components, since no clear difference was observed between the samples.

Considering the data obtained, it is clear that the PE from E25 presents a moderate quality in relation to the phytochemical composition, especially in terms of phenolic acids. Thus, this extract presents more promising, among the extracts obtained, applications for the development of nutraceutical and pharmaceutical products or as a natural food preservative, due to the higher content of phenolic compounds, better antimicrobial activity, as well as the high content of glucosinolates.

### 2.2. Processed Flour (PF)

The characterization of the flours obtained is important to investigate future applications. Table 3 presents the characterization of the processed flours (PFs) obtained using the different solvents tested. The soluble proteins contents in the PFs produced were similar between the treatments, as also observed for the PEs (Table 1). Protein solubility is considered the thermodynamic manifestation of the equilibrium between protein–protein and protein–solvent interactions and is directly related to the physicochemical nature of the protein [36]. Since the solvent composition did not influence the extraction of this compound, it is likely that there were no significant changes in the structural conformations of the protein–protein interaction [37]. Therefore, the retention of part of the proteins in the PFs makes it possible to enhance the value of this by-product in nutritional terms, since its presence makes it viable for use as a raw material for obtaining new products.

As can be seen in Table 3, the PFs presented low glucosinolates content (≤10.64 µmol/g), indicating that the process was effective in removing these compounds, especially when applied to E75. Additionally, the PFs presented low tannin content (≤0.55 mg/g), and E75 resulted in the greatest removal of these compounds. It is worth mentioning that glucosinolates removal must be performed before the use of DM in animal feed, due to digestion dysfunctions and reproductive impairment [7]. Furthermore, tannins are considered antinutritional factors because they decrease the digestibility of proteins, limiting the absorption of this nutrient [38].

Moderate protein content and low content of antinutritional compounds make the PFs potential products for application in pet food, as a secondary source of protein in treats (biscuits, semi-moist formulas, sachets and canned foods) [39]. However, studies on nutritional support, as well as the digestibility and bioavailability of nutrients, should be developed for plant-based products intended for feeding domestic animals [40]. The same benefits can be used for human consumption, for example as a functional ingredient. However, it is important to highlight, in both cases, the need for toxicological evaluation to ensure the safety of the product.

The phenolic contents of the different PFs were also evaluated and presented in Table 3. In general, the PF presented lower levels of phenolic compounds than the PE, indicating high extraction. Although a difference was observed between the values of individual phenolics, the total content of these was slightly influenced by the extract solvent. The main phenolic acids present in the PFs were ferulic, caffeic and cumaric acid, constituting >85% of the total quantified in the products. Ferulic and caffeic acid are also the main phenolic derivatives reported in the composition of extrudates [41], products that were the precursors to the incorporation of plant-based ingredients into pet food formulas [42]. This suggests that the addition of the PF as a total or partial substitute for the cereals used can improve the functional properties and increase the nutritional value of these products. Additionally, caffeic acid has anti-inflammatory and probiotic effects when included in the diet, improving the function of the intestinal microbiota [43].

Regarding the color parameters, L * (brightness) can range from 0 (absolute black) to 100 (absolute white); a * is associated with red–green changes (+a * is red and −a * is green); and b* is associated with yellow–blue changes (+b* is yellow and −b* is blue). The PFs presented slightly light, reddish and yellowish tones, indicated by the value of L* and positive values of a* and b*, respectively, which is an advantage, since darker colors may present greater difficulty of compatibility for food applications [44]. The hue angle (h*) values (73.88° to 76.55°) are in the first trigonometric quadrant, which confirms that the samples’ hues are yellowish-reddish. Additionally, due to the color presented, the PFs can be applied to pet food products, since it is recommended to produce dry dog food with moderate color intensity due to greater acceptability by owners [45]. Furthermore, the color parameters of PFs were compatible with canned food (L* = 55.44, a* = 8.43, and b* = 17.20) [46] and dry pet food (L* = 44.03, a* = 3.21, and b* = 20.57) [47]. The color of the PFs can also be compatible with bakery (bread, cookies, cakes and pasta) and meat products [44].

The difference found between the color parameters of the PFs is possibly associated with the chemical composition, as can be observed in sample E25, which presented the highest luminosity (L*) and lowest a* value among the samples. Szydłowska-Czerniak et al. (2011) [48] determined a positive and significant correlation between L* and the glucosinolate content and an inverse relationship between a* and phenolic compounds of rapeseed varieties.

Due to the lowest content of glucosinolates and tannins and similar values of soluble protein, total phenolics and color parameters, the E75 extraction is considered the most adequate for PF production and these characteristics reinforce that the byproduct studied has great potential to generate high added value and safe products, such as functional foods and pet foods. Therefore, Table 4 presents the results of additional characterization of this PF.

As previously reported PF is considered an adequate source of proteins, being composed mainly by glutelin fraction, followed by albumin and globulin fractions. Extraction conditions can influence the composition and properties of DM proteins, as also can promote the formation of cross-linking between proteins [49]. Thus, it was expected that glutelin was the major fraction of PF, which corresponds to the alkali soluble fraction, remaining after removal of aqueous, ionic and ethanolic fractions. Otherwise, prolamin is soluble in alcohol solutions, and must be mostly removed in the extraction process. Therefore, it was found in the lowest proportion in the PF [50].

Dietary fibers were found in high proportion in the PF (>50%), since this class of compounds, mainly formed by cellulose, hemicellulose, and lignin, are commonly insoluble in ethanolic solutions. This compound is important for pet diets, due to the occurrence of obesity and digestive diseases associated with the consumption of commercial pet foods, that generally had low dietary fiber content [51]. Furthermore, animal’s digestive enzymes cannot digest dietary fiber; thus, the inclusion of the PF in pet foods formulations contribute to a decrease their energetic value, which is interesting for some companies that are intended for weight loss diets [52].

Dietary fibers can also be used as functional additive in pet or human food products and PF can be considered a low-cost source of this nutrient, because the by-products of extractions are usually discarded [53]. In this sense, the functional properties of PF were evaluated (Table 4), to direct its use in future works.

The water solubility index (WAI) of the PF indicates the presence of soluble compounds, such as some proteins, carbohydrates, and soluble dietary fiber, which remained after the extraction process with ethanolic solution (75%). The PF also exhibits water absorption, due the presence of high amounts of proteins and dietary fiber. WAI can increase the water retention in processed foods and contribute to texture stabilization and viscosity [54]. Regarding densities, the value found in this work is within the range (0.40 to 0.59 g/mL) found by Donadelli et al. (2021) [42], whose values did not influence the extrusion process used to produce dry cat food.

## 3. Materials and Methods

### 3.1. Materials

The defatted meal of crambe seeds, with a humidity of 4.58 ± 0.01 wt%, was obtained in a previous study [13], in which oil (~35 wt%) was removed using subcritical propane at 45 °C and 20 bar.

### 3.2. Chemicals and Solvents Used

Ethanol (Química Moderna, Barueri, SP, Brazil, 95%) and distilled water (TE-4007-20, Tecnal, Niort, France) were used as solvents.

To determine the soluble protein content, the following were used: sodium hydroxide (Anidrol, Diadema, SP, Brazil, ≥97.0%), sodium citrate (Synch, New York, NY, USA, ≥99.0%), sodium carbonate (Êxodo Cientifica, Sumaré, SP, Brazil, ≥98.5%), copper sulfate (Anidrol, ≥98.0%), Follin and Ciocalteu’s phenol reagent 2N (Sigma-Aldrich, St. Louis, MO, USA) and bovine albumin as standard (Sigma-Aldrich ≥98.0%).

To determine the total glucosinolate content, sodium tetrachloropalladate (Sigma-Aldrich, ≥98.0%), methanol (HPLC grade, PanReac Applichem, Barcelona, Spain), hydrochloric acid (Anidrol, ≥37%) and sinigrine monohydrated (Sigma-Aldrich, ≥99.0%) were used as reagents. For the quantification of tannins, sulfuric acid (Anidrol, ≥95.0%), methanol (HPLC grade, PanReac Applichem), and vanillin (Sigma-Aldrich, ≥99.0%) were used as reagents, and catechin (Sigma-Aldrich, ≥96.0%) was used as the standard.

Sucrose content was determined using acetonitrile (HPLC grade, Merck, Darmstadt, Germany) and sucrose standard (Sigma Aldrich, >99.0%). For the identification and quantification of phenolic compounds, the following materials were used: ultra-pure water (Milli-Q plus, Induslab, Londrina, SP, Brazil), methanol (HPLC grade, Merck, Darmstadt, Germany), formic acid (Merck, >99.0%,) and chromatographic standards of gallic, trans cinnamic, caffeic, coumaric, ferulic and quercetin acids, all from Sigma-Aldrich (≥98.0%).

In the analyses of antimicrobial activities, sterile water containing 2% dimethyl sulfoxide (Sigma-Aldrich) was used and 2,3,5-triphenyl-tetrazolium chloride (Sigma-Aldrich) at 2.0% was used as a developer. For the cultivation of bacteria and fungus, BHI (brain heart infusion) and Sabouraud broths were used, respectively.

For protein fractions, sodium chloride (Synth, Diadema, Brazil, 98.0%), ethanol (Exodo Científica, 99%), sodium hydroxide (Synth, 98%) and bovine albumin (Signa-Aldrich ≥98.0%) were used. For the determination of dietary fiber, the buffer 2-Morpholinoethanesulfonic acid/ Tris(hydroxymethyl)aminomethane (Neon, Rio de Janeiro, Brazil, 99%) and the dietary fiber determination kit (Merck) were used. In the determination of functional properties, distilled water (TE-4007–20, Tecnal) and soybean oil (Imcopa oils) were used.

### 3.3. Pressurized Liquid Extraction

The apparatus and experimental procedure used in the extraction process was described in detail by Costa et al. [55]. For each extraction, the extractor was filled with ~1 g of DM intercalated with glass beads. The system was operated at 100 bar, adopting 10 min and 30 min for static and dynamic extraction, respectively. During dynamic extraction, the solvent was continuously fed at a flow rate of 1 mL/min. Preliminary tests conducted under these conditions and temperatures of 40, 55 and 70 °C indicated a higher mass yield and content of phenolic compounds in the product obtained at 70 °C, and that temperature was selected to conduct the tests. In the extractions, the amount of ethanol used in the hydroalcoholic solvent varied by 25%, 50% and 75% (by volume) in order to conduct the extractions called E25, E50 and E75, respectively.

After each extraction, the collected liquid extract was concentrated until the complete elimination of the solvent in a vacuum rotary evaporator (MA 120, Marconi, Piracicaba, Brazil). The mass extract yield (MEY) was calculated considering the relationship between the mass of the extract obtained and the initial mass of the sample introduced into the extractor.

Furthermore, processed flour (PF) was obtained from the extractor bed, which, after removal from the extractor, was placed under a Petri dish for washing with ethanol (Anidrol, 70%) and dried in an oven for 1 h at 80 °C (Marconi, MA033/3/E). Figure S1 shows a photo of the PFs obtained, the seeds used and the defatted meal.

### 3.4. Characterization of the Phytochemical Extract

Soluble protein (SP) content was determined as described by Lowry et al. (1951) [56]. The method that uses sodium tetrachloropalladate [57] for color development was selected for the analysis of total glucosinolates and quantification was performed by external standardization using sinigrin. The determination of tannins was carried out according to the methodology that uses vanillin as a reagent, described by Price, Scoyoc and Butler (1978) [58] with some modifications [59]. Briefly, 0.5 mL of extract (diluted in 1% H_2_SO_4_ in methanol) was added to 2.5 mL of vanillin reagent (0.5% (*w*/*v*) vanillin in 2% H_2_SO_4_ in methanol). The mixture was homogenized by vortex and incubated in a water bath at 30 °C for 20 min for subsequent reading of absorbance at 500 nm with a UV/Vis spectrophotometer (Shimadzu UV-1900, Tokyo, Japan).

The extracts were diluted in hydroethanolic solvent (75% ethanol, *v*/*v*) to obtain a concentration of 15.0 mg/mL, filtered through a 0.45 μm membrane and analyzed for sucrose and phenolic contents by high-performance liquid chromatography (HPLC). The sucrose content was determined in a Shimadzu HPLC 20A (Kyoto, Japan), with a refractive index detector (RID-10A) and Aminex HPX-87P column. The chromatographic run was performed at 40 °C, in isocratic mode, using 80% (*v*/*v*) acetonitrile, at 1.0 mL/min for 20 min [60]. The sucrose content was obtained through the standard calibration curve using sucrose at concentrations of 0.6 to 16.0 mg. Figure S2 shows the chromatogram of an analyzed sample of the analytical standard.

The chromatographic separation of phenolic compounds was carried out on a Prominence HPLC (Shimadzu) at 25 °C, using a C-18 column (Shim-pack CLC-ODS (H)™, 25 cm × 4.6 mm × 5 mm). Ultrapure water (A) and methanol (B) acidified with 0.05% and 0.10% (*v*/*v*) formic acid, respectively, were used as mobile phases, at a flow rate of 0.8 mL min^−1^. The elution gradient was used as follows: 5% of B from 0 to 5 min, 20% of B from 5 to 15 min and 80% of B from 15 to 40 min [61]. Detection was performed at 320 nm (ultraviolet detector, SPD-20A). Analytical curves (R^2^ > 0.99) were obtained from phenolic standards (1 to 10 mg/mL). Figure S3 shows the chromatogram of an analyzed sample.

Antimicrobial activity was assessed using the minimum inhibitory concentration (MIC) and minimum bactericidal concentration (MBC), performed using the broth microdilution method in 96-well plates, as described by CLSI [62], with adaptations as required for the experimental model, using *Staphylococcus aureus* (ATCC 12026), *Escherichia coli* (ATCC 25922) and *Candida albicans* (ATCC 10231). The extracts were solubilized in sterile water containing 2% DMSO, obtaining a final concentration of 500 mg/mL. An aliquot of 100 µL of each sample was transferred to microplates containing 100 µL of BHI broth (for bacteria) or Sabouraud broth (for fungi). Then, serial dilutions (1:2) were performed to obtain final concentrations between 250.00 and 0.488 mg/mL. In each well of the microplate, 10 µL of microbial suspensions were added, with concentrations of 1.5 × 10^8^ CFU/mL for bacteria or 1.5 × 10^6^ CFU/mL for fungi. The microplates were then incubated at 35 ± 2 °C for 24 h for bacterial cultures, or at 25 ± 2 °C for 48 h for fungal cultures. The MICs were defined as the lowest concentrations of the extracts that effectively inhibited the growth of the tested microorganisms.

For the determination of the MBC, cultures were plated onto BHI or Sabouraud agar. The plates were subsequently incubated for 48 h. The MBC was identified as the lowest concentration at which no microbial colonies were observed on the agar medium. All experiments were performed in triplicate, and the results are presented as the average of the three trials.

### 3.5. Characterization of Processed Flour

To determine the SP, total glucosinolates and tannins content, the samples were previously prepared and analyzed as specified in Section 3.4. For the soluble proteins content, a NaOH solution (1.25%, *m*/*v*) was added to the sample in a ratio of 1:70 (g/mL) and the solution was subjected to shaking (Marconi, MA 839/A) at 200 rpm for 45 min at 60 °C [63]. In the case of total glucosinolates, 2 mL of 80% (*v*/*v*) methanol was added to the sample (0.1 g) and homogenization was performed for 2 min in a vortex. After incubation at room temperature overnight, the sample was centrifuged (3000 rpm for 4 min) and the supernatant collected [57]. The extraction of tannins from the sample (0.250 g) was performed with 5 mL of H_2_SO_4_ (1%, *m*/*v*) in methanol at 120 rpm and 30 °C for 1 h [59]. The phenolic acids were extracted with water (proportion of 20 mL/g) at 25 °C for 4 h and 150 rpm (Marconi, MA830/A). After this step, the material was centrifuged (Quimis, Diadema, Brazil, Q222TM1) at 3000 rpm for 15 min and the supernatant removed and stored, and the solid was subjected to two new extractions, with the addition of 10 mL of water in each one. The supernatants were combined and stored at −10 °C until analysis.

Color parameters L* (lightness), a* (redness–greenness), and b* (yellowness–blueness) was assessed by a color reader (CR-10, Konica Minolta, Osaka, Japan). The hue angle (h*) values were calculated according to the following equation:

h* (°) = arctan (b*/a*)(1)

To obtain the protein fractions, albumin, globulin, prolamin and glutelin, sequential extraction was performed with the following solvents: water, NaCl 0.9% (*m*/*v*), ethanol 70% (*v*/*v*) and NaOH 0.1 mol/L. For each extraction, a ratio of 1:10 (sample: solvent, *m*/*v*) was used, with shaking at 180 rpm (Shaker Tecnal TE-141) at 25 °C for 30 min. After centrifugation (1512× *g* for 20 min) the supernatant was filtered and its volume made up to 25 mL [64]. The analytical curve was obtained from bovine albumin (0.06 to 0.50 mg/mL) and the results were expressed in g per 100 g of soluble protein [56].

Total, soluble and insoluble dietary fiber content was determined following the gravimetric enzymatic method, 985.29, of the Association of Official Agricultural Chemists—AOAC (2005) [65]. The functional properties were determined based on the procedure described by Seibel and Beléia (2009) [66].

### 3.6. Statistical Analyses

All experiments and analyses were conducted in duplicate (mean ± SD) and the difference between the variables was estimated from the analysis of variance (ANOVA) and Tukey test, adopting *p* ≤ 0.05 as statistically different. For that, the Statistica^®^ 8.0 software was used (StatSoft, Inc., Tulsa, OK, USA).

## 4. Conclusions

This study showed that the defatted meal from crambe seeds is an important source of active compounds and determined for the first time a strategy for valorization of this defatted meal, in which PLE was effectively applied to obtain compounds with potential applications. Based on the composition of the products, it is evident that the process should be conducted using a solvent with 25% ethanol to obtain the phytochemical extract and with 75% ethanol to obtain the processed flour. A phytochemical extract with a moderate content of phenolic compounds (420 mg/100 g), soluble proteins (~23.5 wt%), glucosinolates (259 µmol/g) and tannins (8.8 mg/g) was obtained. An application for the extracts obtained is as an antimicrobial substance, especially against *C. albicans*. Due to the highest removal of glucosinolates and tannins in E75, this extraction is recommended to obtain a PF with high contents of soluble proteins (~26 wt%) and dietary fibers (54.25 g/100 g). This composition was shown to have beneficial functional properties, such as water absorption, which can allow for PF application in pet or human foods formulations. These findings demonstrate the potential of defatted meal from crambe seeds as a viable option for obtaining valuable compounds for functional food, nutraceutical, cosmetic, and pharmaceutical applications due to their composition. Therefore, future research will focus on the evaluation of the toxicity of products obtained, in different concentrations, and applicability tests.

## Figures and Tables

**Table 1:** Effect of solvent composition on obtaining and characterizing the phytochemical extract (PE).

Property		Run		
		E25	E50	E75
MEY (wt%)		23.48 ± 0.30 ^a^	17.68 ± 1.24 ^b^	18.25 ± 0.25 ^b^
Soluble protein (g/100 g)		25.72± 0.12 ^a^	25.76± 0.13 ^a^	24.25± 0.56 ^a^
Total Glucosinolates (µmol/g)		166.87 ± 14.56 ^b^	175.50 ± 3.09 ^b^	258.94 ± 7.4 ^a^
Tannins (mg/g)		5.75 ± 0.14 ^c^	7.54 ± 0.53 ^b^	8.80 ± 0.63 ^a^
Sucrose (mg/100 g)		9.93 ± 0.11 ^b^	11.57 ± 0.0 ^a^	9.29 ± 0.06 ^c^
Phenolic (mg/100 g)	Gallic acid	122.75 ± 4.73 ^a^	97.51 ± 4.23 ^b^	59.72 ± 5.40 ^c^
	*trans*-cinnamic acid	6.31 ± 0.02 ^a^	2.97 ± 0.10 ^b^	1.12 ± 0.03 ^c^
	Caffeic acid	182.45 ± 0.85 ^a^	172.40 ± 6.44 ^b^	146.26 ± 0.64 ^c^
	Cumaric acid	14.46 ± 0.09 ^a^	11.53 ± 0.29 ^b^	7.37 ± 0.17 ^c^
	Ferulic acid	95.46 ± 1.5 ^b^	105.32 ± 3.73 ^a^	61.03 ± 0.47 ^c^
Quercetin (mg/100 g)		0.73 ± 0.01 ^c^	1.01 ± 0.01 ^a^	0.92 ± 0.02 ^b^

MEY: mass extract yield. Means followed by the same letter (in each line) are not statistically different (*p* > 0.05).

**Table 2:** Minimum inhibitory concentration (MIC) and minimum bactericidal concentration (MBC) of phytochemical extract (PE).

Microorganism	Run					
	E25	E50	E75	E25	E50	E75
	MIC (mg/mL)			MBC (mg/mL)		
*E. coli*	125.00 ± 0.0	>250.00	125.00 ± 0.0	>250.00	na	>250.00
*S. aureus*	125.00 ± 0.0	>250.00	125.00 ± 0.0	>250.00	na	>250.00
*C. albicans*	15.62 ± 0.0	15.62 ± 0.0	31.25 ± 0.0	125.00	125.00	125.00

na: not active.

**Table 3:** Effect of solvent composition on the characterization of the processed flour (PF).

Property		Run		
		E25	E50	E75
Soluble protein (g/100 g)		24.94 ± 0.11 ^a^	23.70 ± 0.08 ^a^	26.39 ± 1.36 ^a^
Total Glucosinolates (µmol/g)		10.64 ± 0.67 ^a^	11.76 ± 0.37 ^a^	3.57 ± 0.19 ^b^
Tannins (mg/g)		0.55 ± 0.08 ^a^	0.57 ± 0.06 ^a^	0.35 ± 0.02 ^b^
Phenolic (mg/100 g)	Gallic acid	nd	0.17 ± 0.01 ^b^	2.33 ± 0.22 ^a^
	*trans*-cinnamic acid	1.27 ± 0.06 ^a^	0.88 ± 0.03 ^b^	0.56 ± 0.03 ^c^
	Caffeic acid	6.24 ± 0.01 ^a^	6.24 ± 0.01 ^a^	6.23 ± 0.05 ^a^
	Cumaric acid	5.25 ± 0.06 ^a^	4.90 ± 0.17 ^b^	4.99 ± 0.09 ^ab^
	Ferulic acid	9.73 ± 0.24 ^a^	7.11 ± 0.38 ^c^	8.18 ± 0.43 ^b^
Quercetin (mg/100 g)		1.23 ± 0.07 ^a^	0.93 ± 0.05 ^b^	0.82 ± 0.003 ^b^
Color parameters	L*	58.29 ± 0.23 ^a^	56.35 ± 0.17 ^c^	57.77 ± 0.10 ^b^
	a*	4.92 ± 0.11 ^c^	5.21 ± 0.04 ^b^	5.89 ± 0.08 ^a^
	b*	20.58 ± 0.19 ^b^	21.51 ± 0.11 ^a^	20.38 ± 0.38 ^b^
	h* (°)	76.55	76.38	73.88

nd: not detected. Means followed by the same letter (in each line) are not statistically different (*p* > 0.05).

**Table 4:** Protein fractions, dietary fiber content and functional properties of processed flour (PF ^1^).

Protein fractions (g/100 g soluble protein)			
Albumin		5.02 ± 0.77	
Globulin		5.22 ± 0.21	
Prolamin		2.64 ± 0.18	
Glutelin		8.97 ± 0.59	
Dietary fiber (g/100 g)			
Total	54.25 ± 4.89		
Functional properties			
Apparent density (g/mL)			0.47 ± 0.01
Water Solubility Index (%)			4.00 ± 0.01
Water Absorption Index (%)			4.27 ± 0.01

^1^ obtained from run E75.

## Data Availability

The datasets supporting the conclusions of this article are included within the manuscript.

## Supplementary Materials

The following supporting information can be downloaded at https://www.mdpi.com/article/10.3390/plants14030326/s1; Figure S1. Photos of the crambe seeds, defatted meal and processed flours. Figure S2. HPLC chromatogram of (a) sample and (b) sucrose standard. Figure S3. HPLC chromatogram of the profile of phenolic compounds present in the phytochemical extract: (1) gallic acid, (2) caffeic acid, (3) coumaric acid, (4) ferulic acid, (5) *trans*-cinnamic acid and (6) quercetin.
