# Supplementary material for: Application of the Biorefinery Concept in the Processing of Crambe (Crambe abyssinica Hochst) Seed Defatted Meal in a Pressurized Medium
Source: Plants (Basel). 2025 Jan 22;14(3):326. doi: 10.3390/plants14030326 (PMC11821204; doi:10.3390/plants14030326)
Supplement: Supplementary file 1 [file plants-14-00326-s001.zip › plants-3389602-supplementary.pdf]

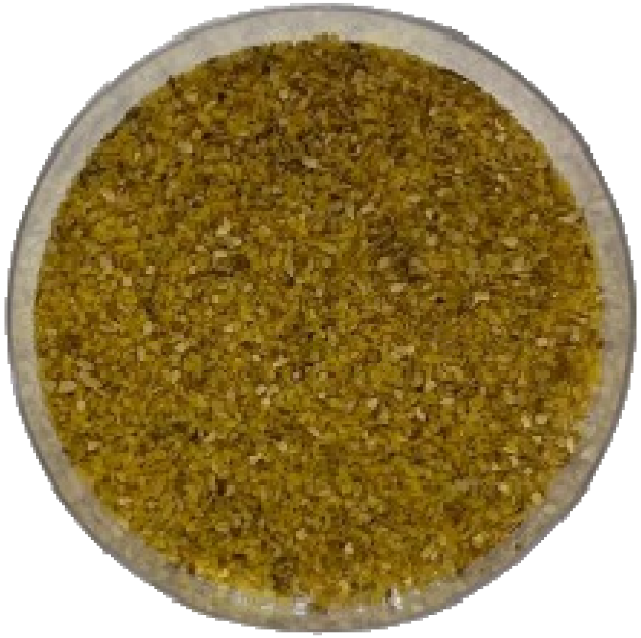

Crambe seeds

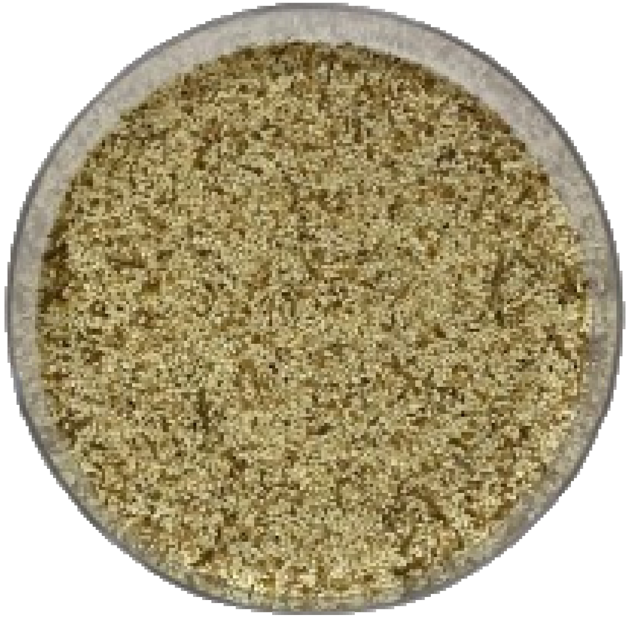

Defatted meal

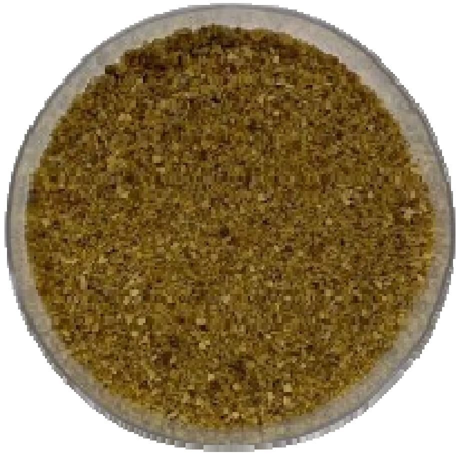

Processed flour (run E25)

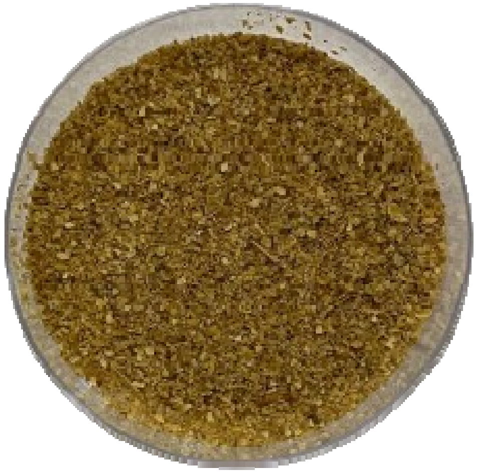

Processed flour (run E50)

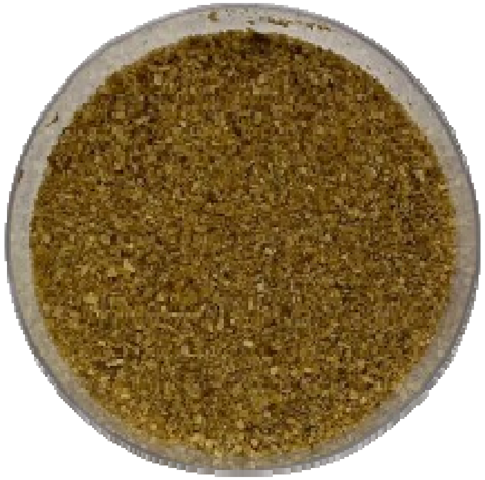

Processed flour (run E75)

Figure S1. Photos of the crambe seeds, defatted meal and processed flours.

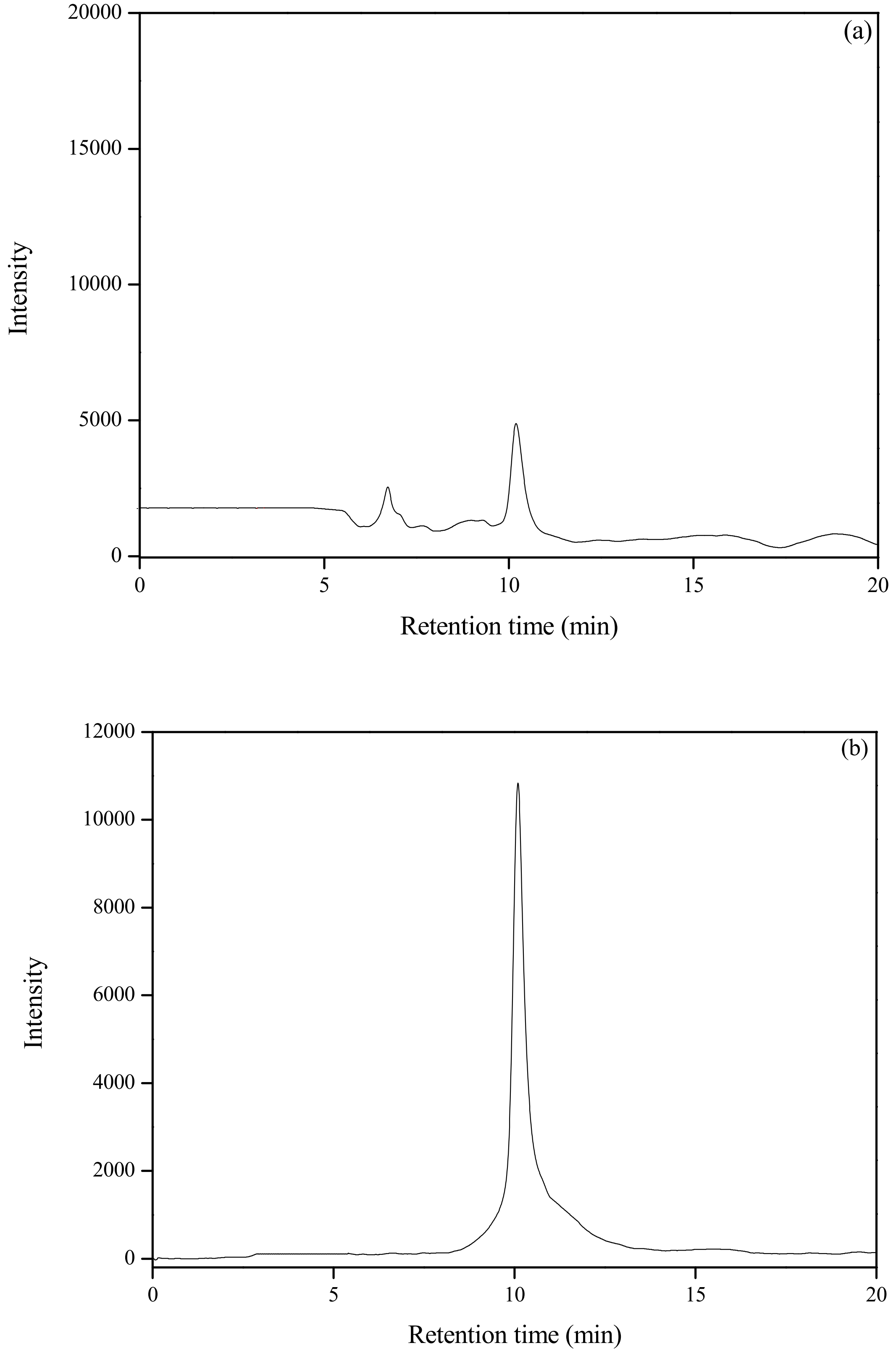

Figure S2. HPLC chromatogram of (a) sample and (b) sucrose standard.

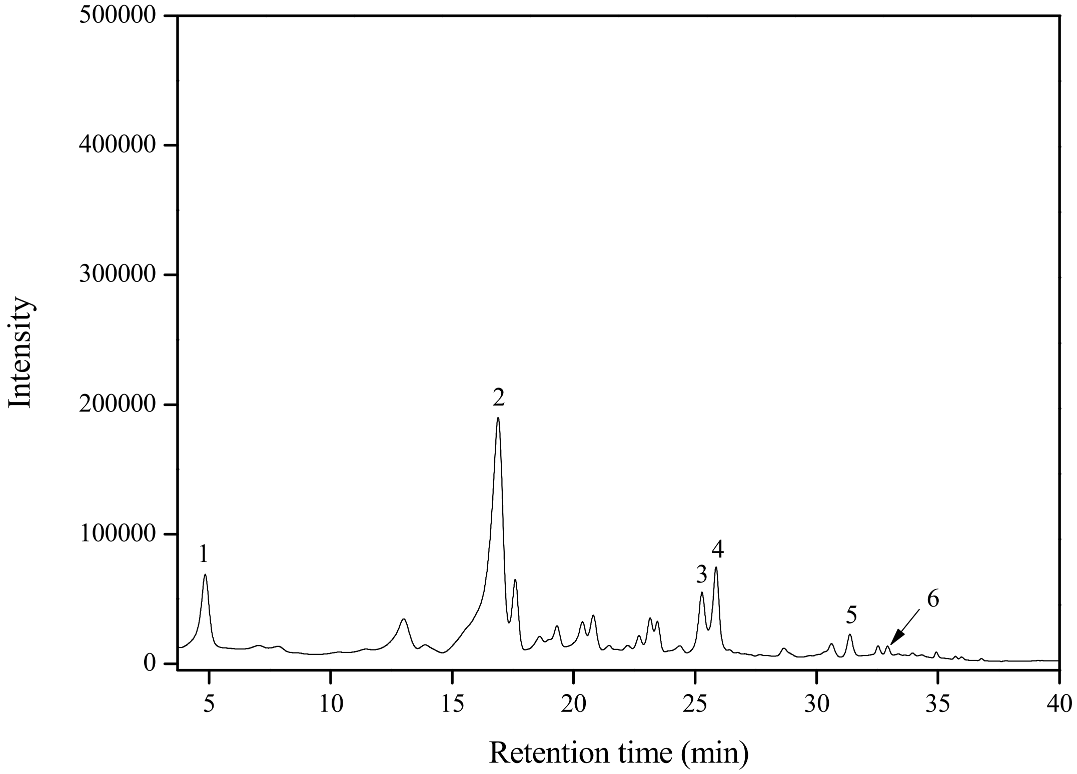

Figure S3. HPLC chromatogram of the profile of phenolic compounds present in the phytochemical extract: (1) gallic acid, (2) caffeic acid, (3) coumaric acid, (4) ferulic acid, (5) *trans*-cinnamic acid and (6) quercetin.
